# *RETSAT* Mutation Selected for Hypoxia Adaptation Inhibits Tumor Growth

**DOI:** 10.3389/fcell.2021.744992

**Published:** 2021-11-04

**Authors:** Xiulin Jiang, Yaomei He, Qiushuo Shen, Lincan Duan, Yixiao Yuan, Lin Tang, Yulin Shi, Baiyang Liu, Haoqing Zhai, Peng Shi, Cuiping Yang, Yongbin Chen

**Affiliations:** ^1^ Key Laboratory of Animal Models and Human Disease Mechanisms of Chinese Academy of Sciences and Yunnan Province, Kunming Institute of Zoology, Kunming, China; ^2^ Kunming College of Life Science, University of Chinese Academy of Sciences, Beijing, China; ^3^ The Third Affiliated Hospital of Kunming Medical University, Kunming, China; ^4^ State Key Laboratory of Genetic Resources and Evolution, Kunming Institute of Zoology, Chinese Academy of Sciences, Kunming, China; ^5^ Center for Excellence in Animal Evolution and Genetics, Chinese Academy of Sciences, Kunming, China

**Keywords:** retinol saturase (RETSAT), hypoxia adaptation, evolution, Pin1, skin cutaneous melanoma (SKCM)

## Abstract

Hypoxia occurs not only in natural environments including high altitude, underground burrows and deep sea, but also in human pathological conditions, such as hypoxic solid tumors. It has been well documented that hypoxia related signaling pathway is associated with a poor clinical outcome. Our group has recently identified multiple novel genes critical for solid tumor growth comparing the genome-wide convergent/parallel sequence evolution of highland mammals. Among them, a single mutation on the retinol saturase gene (*RETSAT*) containing amino acid switch from glutamine (Q) to arginine (R) at the position 247 was identified. Here, we demonstrate that RETSAT is mostly downregulated in multiple types of human cancers, whose lower expression correlates with worse clinical outcome. We show that higher expression of RETSAT is positively associated with immune infiltration in different human cancers. Furthermore, we identify that the promoter region of *RETSAT* is highly methylated, which leads to its decreased expressions in tumor tissues comparing to normal tissues. Furthermore, we show that RETSAT knockdown promotes, while its overexpression inhibits, the cell proliferation ability of mouse embryonic fibroblasts (MEFs) and B16 *in vitro*. In addition, the mice carrying homozygous Q247R mutation (RETSATR/R) is more resistant to xenograft tumor formation, as well as DMBA/TPA induced cutaneous keratinocyte carcinoma formation, compared to littermate wild-type (RETSATQ/Q) mice. Mechanistic study uncovers that the oncogenic factor, the prolyl isomerase (PPIase) Pin1 and its related downstream signaling pathway, were both markedly repressed in the mutant mice compared to the wild-type mice. In summary, these results suggest that interdisciplinary study between evolution and tumor biology can facilitate identification of novel molecular events essential for hypoxic solid tumor growth in the future.

## Introduction

High altitude is one of the most extreme environments worldwide, and mammals living in high altitude evolve adaptation traits including respiratory, cardiovascular, and metabolic systems compared to reciprocal lowlanders (Brutsaert, 2008; Scheinfeldt et al., 2012; Rademaker et al., 2014; Storz and Scott, 2021). The genetic signals after hypoxia positive selection are the major factors contributing to the hypoxia tolerant physiological traits, some of which have been indicated to be critical for transcriptional regulation under hypoxic conditions (Beall et al., 2010; Yi et al., 2010). Hemoglobin levels and oxygen saturation in the blood are two physiological characteristics important for oxygen sense, which are thoroughly studied in highlanders. Hemoglobin concentration is elevated, but oxygen saturation is reduced in high-altitude Andean populations, compared to African population as well as other low-altitude populations (Beall et al., 2002).

Hypoxia occurs not only in natural environments including high altitude, underground burrows and deep sea, but also in human pathological conditions, such as diabetes and hypoxic solid tumors (De Bels et al., 2011; Cheviron and Brumfield, 2012; Yang et al., 2020; Storz and Scott, 2021). However, hypoxia cannot be simply defined by a fixed oxygen concentration, since some tissues function normally at 5% oxygen equivalent to normoxia, and others as low as 1% oxygen (Schödel and Ratcliffe, 2019). Hypoxia related signaling molecules have been documented to function as a major regulator during tumorigenesis (Kang et al., 2018; Xiong et al., 2020). These factors include hypoxia-inducible factors (HIFs), the von Hippel–Lindau (VHL) and prolyl hydroxylases (PHD1/2/3 or so-called EglN1/2/3, respectively) of the 2-oxoglutarate (or α-ketoglutarate) dioxygenase super-family (Schödel and Ratcliffe, 2019), which provide valuable therapeutic targets for various types of human cancers (Lee et al., 2020). Increasing evidence has revealed that hypoxic solid tumors are not sensitive to clinical treatment due to reduced reactive oxygen species (ROS) and DNA damage in the case of ionizing radiation and certain chemotherapies (Brustugun, 2015). However, it is important to note that hypoxia in solid tumors is not completely the same as that in high altitude. For example, some hypoxic tumor regions have near 0% oxygen, while the oxygen level will only drop to ∼60% of sea level at ∼4,000 m highland.

Based on the similarities between high altitude hypoxia adaptation and hypoxic solid tumors, we have developed an interdisciplinary study to identify novel biomarkers involved in tumorigenesis. For examples, we did parallel large-scale genomic data generated from Tibetan domestic mammals and corresponding lowland species, and identified multiple hypoxia adaptation selected genes including YTHDF1 and C10orf67 (Shi et al., 2019; Wu et al., 2020). Furthermore, we found that YTHDF1 expression is decreased in highland mammals compared to lowlanders, which promotes non-small cell lung cancer (NSCLC) progression by activating the translational efficiency of m6A modified CDK2 and CDK4 mRNAs (Shi et al., 2019). Recently, we uncovered that the retinol saturase gene (*RETSAT*) contains a single parallel amino acid change from glutamine (Q) to arginine (R) at position 247 in QTP (Qinghai-Tibet Plateau) mammals (Xu et al., 2021). RETSAT has been identified to be an NADH/NADPH- dependent oxidoreductase, which is highly expressed in liver, adipose tissue and kidney (Moise et al., 2004). Previous studies have shown that RETSAT saturates the 13-14 double bond of all-trans-retinol to produce all-trans-13-14-dihydroretinol, a product important for vitamin A metabolism, which then regulates lipid metabolism, and production of reactive oxygen species (Moise et al., 2005). Furthermore, RETSAT was uncovered to modulate lipid metabolism and the production of reactive oxygen species (ROS) (Pang et al., 2017). RETSAT promotes adipogenesis and is downregulated in obesity by suppressing PPARγ and retinoid X receptor α (RXRα) responses (Ziouzenkova et al., 2007; Schupp et al., 2009).

Previous studies demonstrated that all-trans retinoic acid (ATRA), the active metabolite of vitamin A, could be used for the treatment of acute promyelocytic leukemia (APL) (Gottardi et al., 2020). Another study showed that all-trans retinoic acid stealth liposomes prevent the metastasis of breast cancer and glioblastoma tumor growth (Li et al., 2011; Mirani et al., 2019). However, whether or not RETSAT play any role in human cancers is still unknown. In this study, we decided to verify the expression and mutation patterns of RETSAT in various human cancers, and compared the xneograft or chemical induced-tumor formation in RETSAT mutant and wild-type mice.

## Results

### RETSAT Expression Patterns in Different Types of Human Cancers

**FIGURE 1:**
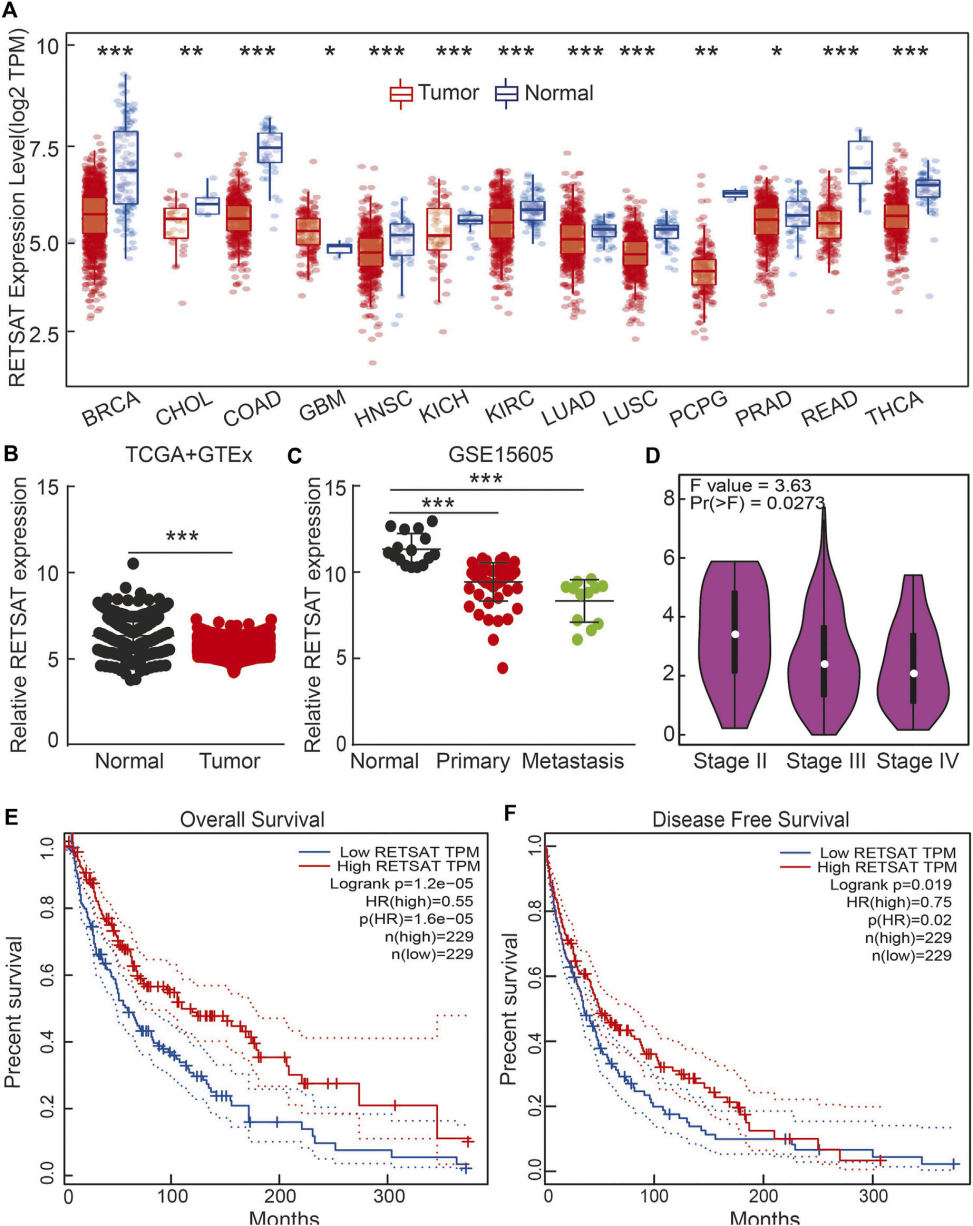
RETSAT expression patterns in human cancers. **(A)** RETSAT expression of different types of human cancers using the TIMER database. Red: tumors; blue: normal tissues. **(B)** The expression pattern of RETSAT, comparing the profiles from SKCM in (TCGA) and GTEx (Genotype-Tissue Expression, mostly for normal tissues). **(C,D)** The expression patterns of RETSAT in SKCM using the GEO databases and the GEPIA database **(D)**. **(E)** The overall survival (OS: E) and the disease-free survival (DFS: F) tests for RETSAT in SKCM in GEPIA database. *p < 0.05, **p < 0.01, ***p < 0.001.

We firstly examined the mRNA expression patterns of RETSAT in different types of human cancers using the Tumor Immune Estimation Resource (TIMER) online database (Li et al., 2017). The consistent lower expression of RETSAT was observed in BRCA (Breast invasive carcinoma), CHOL (Cholangiocarcinoma), COAD (Colon adenocarcinoma), HNSC (Head and Neck squamous cell carcinoma), KICH (Kidney Chromophobe), KIRC (Kidney renal clear cell carcinoma), LUAD (Lung adenocarcinoma), LUSC (Lung squamous cell carcinoma), PCPG (Pheochromocytoma and Paraganglioma), PRAD (Prostate adenocarcinoma), READ (Rectum adenocarcinoma) and THCA (Thyroid carcinoma) compared with the corresponding normal tissues (Figure 1A). The above results showed that RETSAT expression is commonly decreased in most human cancers, indicating that RETSAT may function as a tumor suppressor.

Based on the fact that mammals living in high altitude are exposed to both hypoxic and intensive ultra violet conditions, we decided to validate the expression pattern of RETSAT in skin cutaneous melanoma (SKCM), and identified lower RETSAT transcripts in cancerous tissues than that in normal skin tissues, by using the gene expression profile from TCGA and GETx dataset (Figure 1B). Consistently, we also verified the downregulation of RETSAT in tumor tissues in the GEO database (Figure 1C). The correlation between RETSAT and SKCM tumor stage was further tested using the GEPIA online tool (Tang et al., 2017). We found that lower expressions of RETSAT transcripts correlate with higher tumor stages (Figure 1D). In line with this finding, we revealed that patients with SKCM containing higher RETSAT expression exhibited longer overall survival (OS) and disease-free survival (DFS) time, compared to SKCM patients with lower RETSAT expression levels (Figures 1E,F). In addition, we identified many RETSAT mutations in SKCM by applying the cBioPortal web resource (Table 1) (Gao et al., 2013). The above results suggest that RETSAT is decreased in SKCM, indicating its inhibitory role during SKCM tumor progression.

**TABLE 1:** RETSAT mutations in SKCM determined by cBioportal database.

Cancer type	Change	Mutation type	Copy change	Mut sample
Cutaneous Melanoma	*G536R*	Missense	Gain	35
Cutaneous Melanoma	*A533V*	Missense	Gain	35
Cutaneous Melanoma	*G536R*	Missense	Gain	110
Cutaneous Melanoma	*A533V*	Missense	Gain	110
Cutaneous Melanoma	*P270T*	Missense	Diploid	154
Acral Melanoma	*G536R*	Missense	Diploid	248
Acral Melanoma	*A533V*	Missense	Diploid	248
Cutaneous Melanoma	*S601F*	Missense	Diploid	313
Cutaneous Melanoma	*S601F*	Missense	ShallowDel	322
Cutaneous Melanoma	*S601F*	Missense	ShallowDel	325
Melanoma	*S492F*	Missense	Diploid	346
Cutaneous Melanoma	*P215L*	Missense	Diploid	389
Cutaneous Melanoma	*P215Q*	Missense	Diploid	421
Melanoma	W146*	Nonsense	ShallowDel	443
Cutaneous Melanoma	W146*	Nonsense	ShallowDel	448
Cutaneous Melanoma	*L180F*	Missense	ShallowDel	499
Cutaneous Melanoma	*V258A*	Missense	Diploid	510
Cutaneous Melanoma	*P292T*	Missense	Diploid	510
Cutaneous Melanoma	*V258A*	Missense	Diploid	517
Cutaneous Melanoma	*P292T*	Missense	Diploid	517
Cutaneous Melanoma	*V258A*	Missense	Diploid	517
Cutaneous Melanoma	*P292T*	Missense	Diploid	517
Cutaneous Melanoma	*G536R*	Missense	Diploid	525
Cutaneous Melanoma	*A533V*	Missense	Diploid	525
Cutaneous Melanoma	*L180F*	Missense	Diploid	528
Cutaneous Melanoma	*L180F*	Missense	Diploid	576
Cutaneous Melanoma	*L3H*	Missense	ShallowDel	579
Melanoma	*L3H*	Missense	ShallowDel	582
Cutaneous Melanoma	*L3H*	Missense	ShallowDel	587
Melanoma	*S34F*	Missense	ShallowDel	743
Cutaneous Melanoma	*M420I*	Missense	Diploid	779
Cutaneous Melanoma	*P292S*	Missense	ShallowDel	854
Melanoma	*S34F*	Missense	ShallowDel	855
Cutaneous Melanoma	*R133C*	Missense	Diploid	867
Cutaneous Melanoma	*P292S*	Missense	Diploid	869
Cutaneous Melanoma	*R482W*	Missense	ShallowDel	949
Cutaneous Melanoma	*R482W*	Missense	Diploid	961
Cutaneous Melanoma	*R482W*	Missense	Diploid	966
Cutaneous Melanoma	*P292S*	Missense	Diploid	1,005
Cutaneous Melanoma	*G124W*	Missense	Diploid	1,088
Cutaneous Melanoma	*V54I*	Missense	ShallowDel	1,354
Cutaneous Melanoma	*L315I*	Missense	Diploid	1,369
Cutaneous Melanoma	G172*	Nonsense	Diploid	1,436
Cutaneous Melanoma	*P168H*	Missense	Diploid	1,445
Skin Cancer	*P455S*	Missense	ShallowDel	1,521
Cutaneous Melanoma	*W450C*	Missense	ShallowDel	1,610
Cutaneous Melanoma	*G250W*	Missense	ShallowDel	1,630
Cutaneous Melanoma	*P448S*	Missense	Gain	1,718
Cutaneous Melanoma	*P305H*	Missense	ShallowDel	1,749
Cutaneous Melanoma	*Q418*	Splice	ShallowDel	1,804
Skin Cancer	*P292S*	Missense	ShallowDel	1,806
Skin Cancer	*S60L*	Missense	ShallowDel	1,836
Cutaneous Melanoma	*P506S*	Missense	ShallowDel	1,901
Cutaneous Melanoma	Y176Tfs*20	FS del	ShallowDel	1,937
Cutaneous Melanoma	*P506S*	Missense	Gain	1,938
Cutaneous Melanoma	*P506S*	Missense	Gain	1,967
Cutaneous Melanoma	R164*	Nonsense	Diploid	2,068
Melanoma	G204*	Nonsense	Diploid	2,156
Cutaneous Melanoma	*T388I*	Missense	ShallowDel	3,224
Cutaneous Melanoma	*P168S*	Missense	ShallowDel	3,330
Skin Cancer	*P465L*	Missense	ShallowDel	3,403
Cutaneous Melanoma	*P152S*	Missense	ShallowDel	3,536
Cutaneous Melanoma	*P233L*	Missense	ShallowDel	3,843
Melanoma	*F25Y*	Missense	Diploid	5,023
Cutaneous Melanoma	*S238F*	Missense	Diploid	5,836
Cutaneous Melanoma	R293*	Nonsense	Diploid	7,335
Cutaneous Melanoma	*H207Y*	Missense	Diploid	15,530
Cutaneous Melanoma	*H207Y*	Missense	Diploid	15,834

Notes: *p < 0.05, **p < 0.01, ***p < 0.001.

### The Promoter Region of *RETSAT* is Highly Methylated in Multiple Tumors

**FIGURE 2:**
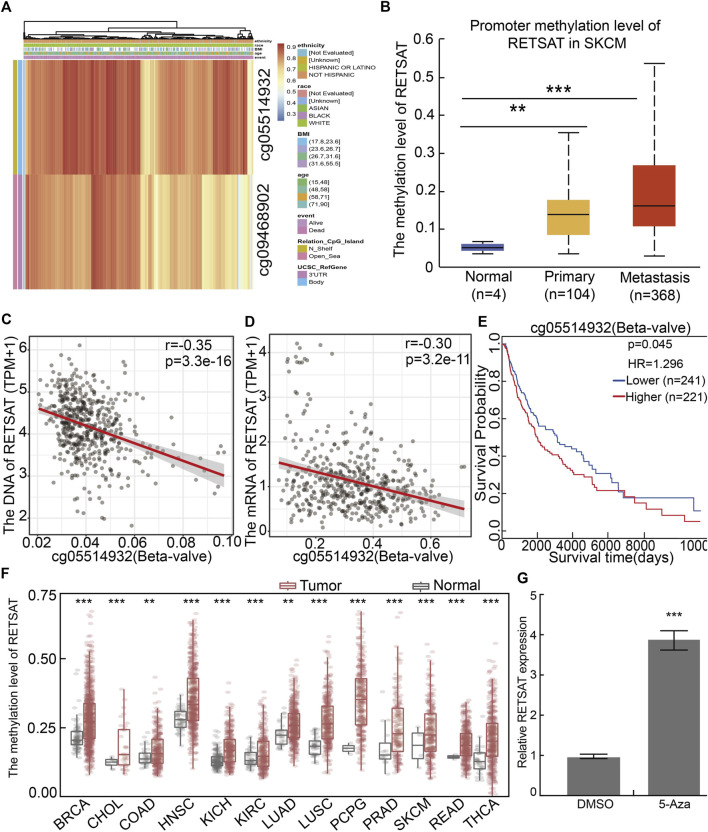
The methylation pattern in RETSAT promoter. **(A)** Critical methylation sites within RETSAT promoter region were indicated in the heatmap. The gradient color bars indicate the different methylation levels of the two methylation sites (cg05514932, cg09468902) within RETSAT promoter region in each SKCM sample, the browner the color is, the higher methylation level is. **(B)** The methylation level in RETSAT promoter region positively correlates with SKCM stages in ualcan database. **(C,D)** Both the DNA **(C)** and mRNA **(D)** expressions of RETSAT negatively correlate with the methylation level using SMART database. **(E)** Kaplan-Meier analysis of OS (overall survival) considering RETSAT methylation level using the TCGA SKCM dataset. **(F)** The methylation level of RETSAT in different types of human cancers compared with that in normal tissues using SMART database. **(G)** The relative expression of RETSAT mRNA in B16 cells after 5-azacytidine treatment, examine by Real time RT-PCR assay. *p < 0.05, **p < 0.01, ***p < 0.001.

To further elucidate the mechanism by which RETSAT is commonly downregulated in tumors, we firstly examined the methylation status of *RETSAT* promoter region, which is major cause for repressing gene expression in tumors. By using the methSurv analysis (Modhukur et al., 2018), we found that there are many methylation sites in the promoter region of *RETSAT*, and the differential methylation regions were indicated in the heatmaps (Figure 2A). Importantly, by using the shiny methylation analysis resource tool (SMART) analysis (Li et al., 2019) we uncovered that the methylation of RETSAT was significantly higher in SKCM cancerous tissues compared to that in normal tissues (Figure 2B). Consistently, we found that the methylation levels on the specific methylation site (cg05514932) within *RETSAT* promoter region negatively correlated with its expression in SKCM (Figures 2C,D). Furthermore, we showed that the elevated methylation levels on cg05514932 site correlates with worse OS in the TCGA-SKCM cohorts, using the methSurv dataset (Figure 2E). Therefore, we hypothesized that the highly methylation in *RETSAT* promoter DNA fragment might cause its decreased expressions in multiple types of human cancers, including SKCM (Figure 2F). In addition, we treated B16 cells with 5-Azacytidine, the specific inhibitor for DNA methylases (Christman, 2002), and revealed that *RETSAT* mRNA expression level was dramatically increased (Figure 2G)*.*

### RETSAT Expression Correlates With Immune Infiltration in SKCM

**FIGURE 3:**
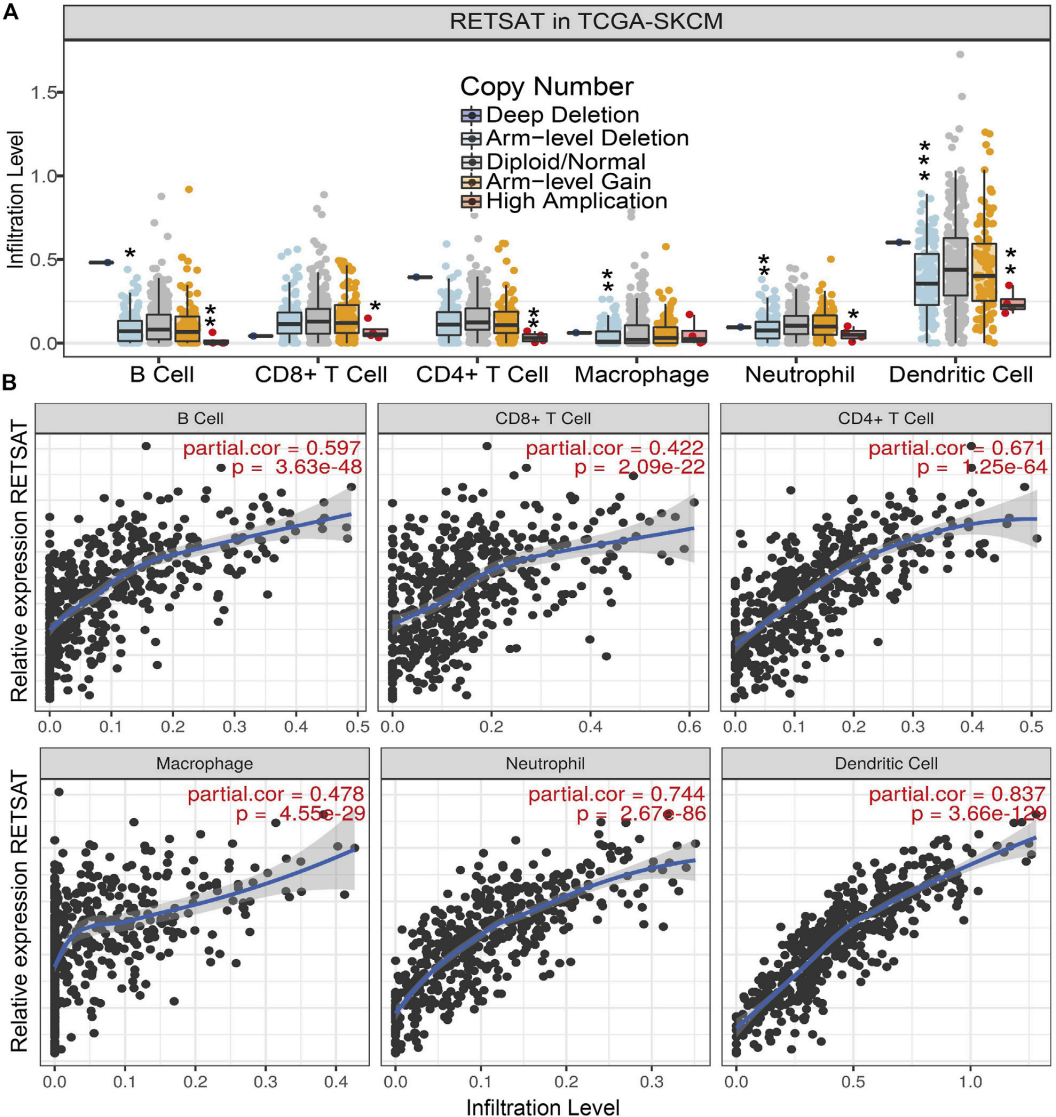
RETSAT correlates with immune cell infiltration in tumors. **(A)** The infiltration levels of various immune cells correlate with RETSAT mutations in SKCM using the TIMER database. **(B)** The correlation of RETSAT expression level with B cell, CD8^+^ T cell, CD4^+^ T cell, macrophage, neutrophil or dendritic cell infiltration level in SKCM analyzed by TIMER database. *p < 0.05, **p < 0.01, ***p < 0.001.

Since RETSAT might function as a tumor suppressor and immune infiltration was considered as a promising independent prognostic factor in cancers, we next used the TIMER database to investigate the correlation between RETSAT expression and immune infiltration (Li et al., 2017). Specifically, *RETSAT* CNV significantly correlates with infiltrating levels of B cells, CD8^+^ T cells, macrophages, neutrophils and dendritic cells (Figure 3A). Next, we analyzed the correlation between RETSAT expression and six types of infiltrating immune cells, including B cells, CD8^+^ T cells, CD4^+^ T cells, macrophages, neutrophils and dendritic cells. The results demonstrated that the expression level of RETSAT was positively correlated with the infiltration level of B cells (*r* = 0.597, *p* = 3.63e-48), CD8^+^ T cells (*r* = 0.422, *p* = 2.09e-22), CD4^+^ T cells (*r* = 0.671, *p* = 1.25e-64), macrophages (*r* = 0.478, *p* = 4.55e-29), neutrophils (*r* = 0.744, *p* = 2.67e-86), and dendritic cells (*r* = 0.837, *p* = 3.66e-129) in SKCM (Figure 3B). Next, we used TISIDB database to further explore the relationship between RETSAT expression level and immunostimulators and immunoinhibitors, respectively in SKCM (Ru et al., 2019). We found that RETSAT was mostly positively, but negatively, associated with the expression of immunostimulators and the immunoinhibitors, respectively (Tables 2, 3). These results strongly implicate that RETSAT could serve as a key regulator for tumor immune infiltration in SKCM.

**TABLE 2:** The correlation analysis between RETSAT and the expression of immunostimulators in SKCM validated by TIMER database.

Immunostimulators	*r*	*p*	Immunostimulators	*r*	*p*
C10ORF54	0.723	***	TNFRSF9	0.845	***
CD27	0.807	***	KLRC1	0.658	***
CD28	0.804	***	KLRK1	0.74	***
CD40	0.668	***	LTA	0.81	***
CD40LG	0.755	***	MICB	0.355	***
CD48	0.823	***	NT5E	0.183	***
CD70	0.436	***	TMEM173	0.253	***
CD80	0.831	***	TMIGD2	0.609	***
CD86	0.881	***	TNFRSF13B	0.685	***
CXCL12	0.699	***	TNFRSF13C	0.411	***
CXCR4	0.694	***	TNFRSF17	0.668	***
ENTPD1	0.516	***	TNFRSF18	0.586	***
ICOS	0.778	***	TNFSF14	0.756	***
ICOSLG	0.532	***	TNFRSF4	0.491	***
IL2RA	0.809	***	TNFRSF8	0.684	***

Notes: *p < 0.05, **p < 0.01, ***p < 0.001.

**TABLE 3:** The correlation analysis between RETSAT and the expressions of immunoinhibitors in SKCM validated by TIMER database.

Immunoinhibitors	*r*	*p*
ADORA2A	−0.295	***
BTLA	−0.199	***
CD160	−0.252	***
CD244	−0.133	***
CD96	−0.248	***
CSF1R	−0.258	***
HAVCR2	−0.19	***
IDO1	−0.268	***
IL10	−0.157	***
KDR	−0.203	***
LAG3	−0.15	***
LGALS9	−0.15	***
PDCD1	−0.116	***
PDCD1LG2	−0.264	***
TIGIT	−0.188	***

Notes: *p < 0.05, **p < 0.01, ***p < 0.001.

### RETSAT Q247R Mutation Inhibits Tumor Growth *in vitro* and *in vivo*

**FIGURE 4:**
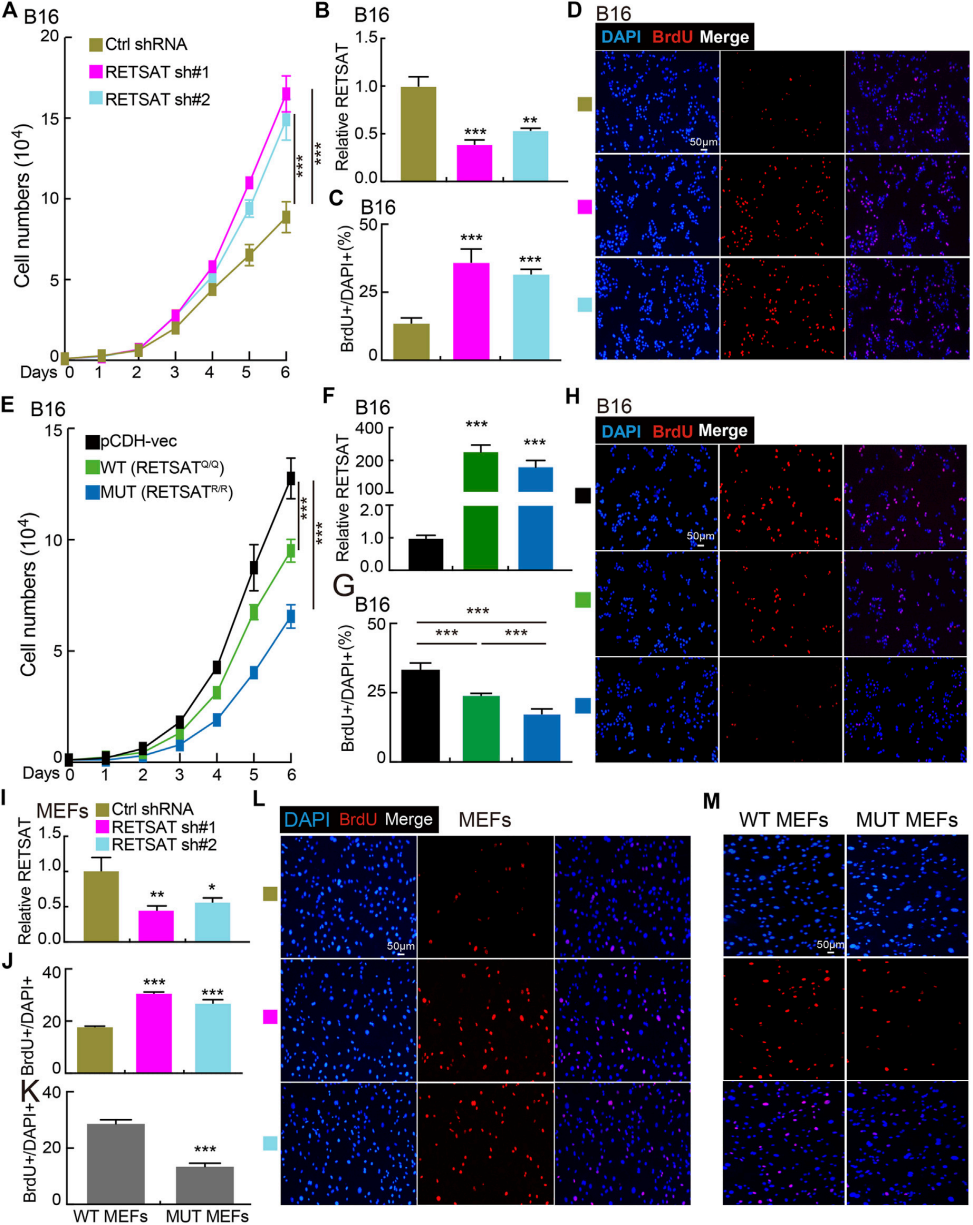
RETSAT knockdown promotes cell proliferation. **(A)** Knockdown of RETSAT promotes B16 cell growth examined by growth curve assay. **(B)** RETSAT targeting shRNA knockdown efficiency in B16 cells was verified by Real-time RT-PCR assay, scramble shRNA was used as control. sh#1 = shRNA#1, sh#2 = shRNA#2, Ctrl = control. **(C,D)** Knockdown of RETSAT promotes B16 cell proliferation examined by BrdU incorporation assay, **(C)** is the quantification data for **(D)**. Scale bar = 50 μm. **(E)** Overexpression of RETSAT inhibits B16 cell proliferation examined by growth curve assay. **(F)** RETSAT overexpression efficiency in B16 cells was verified by Real-time RT-PCR assay. **(G,H)** RETSAT overexpression inhibits B16 cell proliferation examined by BrdU incorporation assay, **(G)** is the quantification data for **(H)**. Scale bar = 50 μm. **(I)** RETSAT targeting shRNA knockdown in wild-type MEFs was verified by Real-time RT-PCR assay. **(J,L)** RETSAT inhibition promotes cell proliferation in MEFs examined by BrdU incorporation assay, **(L)** is the quantification data for **(J)**. Scale bar = 50 μm. **(K,M)** RETSATR/R mutant MEFs exhibit higher proliferation rate compared to wild type RETSATQ/Q MEFs, **(M)** is the quantification data for **(L)**. Scale bar = 50 μm. WT = wild type (RETSATQ/Q). MUT = mutant (RETSATR/R).*p < 0.05, **p < 0.01, ***p < 0.001.

To examine the functional role of RETSAT in SKCM cells, RETSAT was inhibited by two independent lenti-viral shRNAs in B16 cells and mouse embryonic fibroblasts (MEFs), and the knockdown and overexpression efficiencies were verified by Real-time RT-PCR, cell line expressing scramble shRNA was used as control. As expected, RETSAT knockdown promoted the cell proliferation ability of B16 and MEFs (Figures 4A–D,I,J,L), while RETSAT overexpression led to the opposite effect (Figures 4E–H,K,M). Taken together, these data suggest that RETSAT functions as a tumor suppressor in SKCM.

**FIGURE 5:**
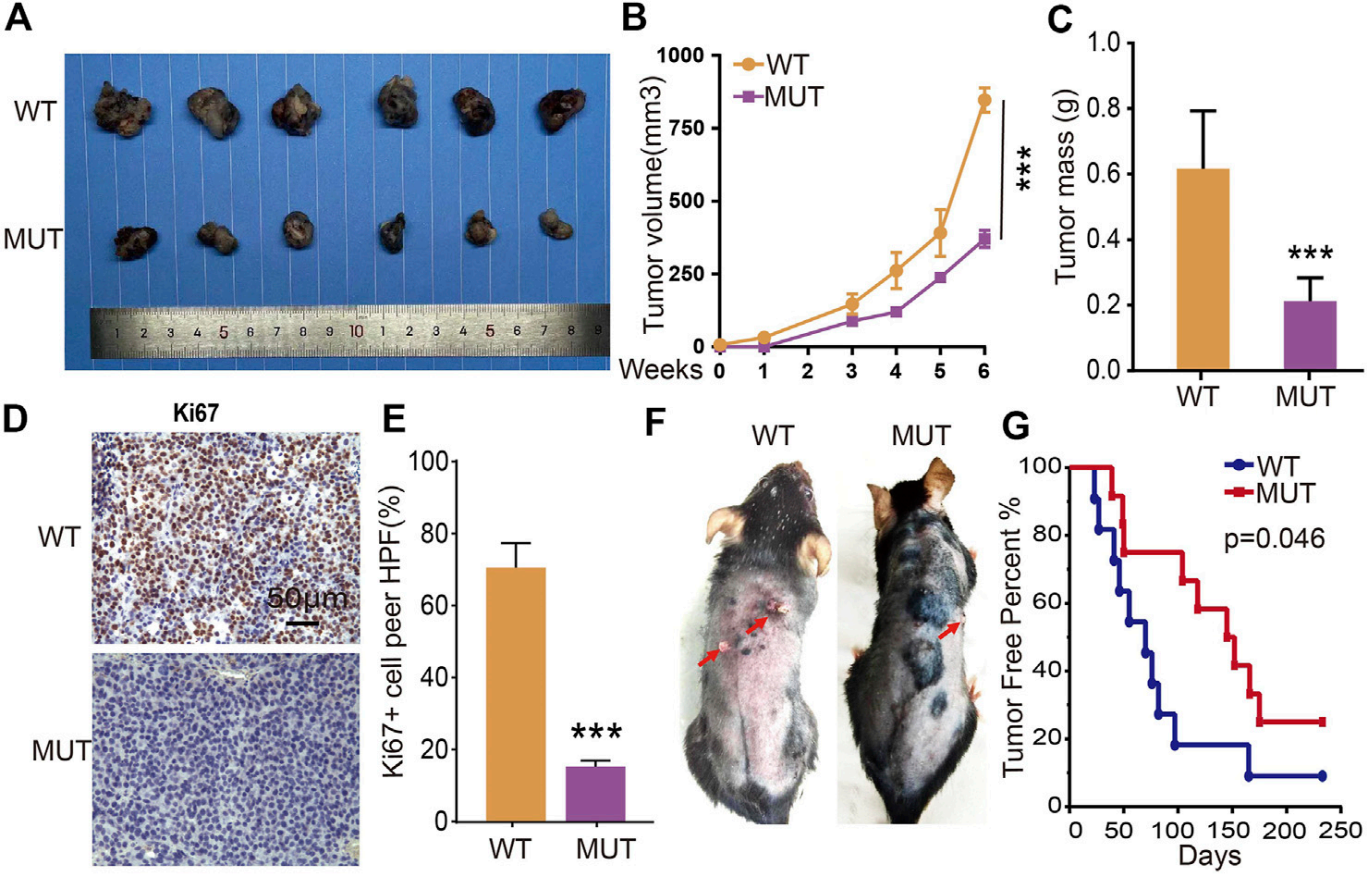
RETSAT (Q247R) mutation inhibits tumor growth *in vivo*. **(A–C)** RETSAT mutation inhibits xenograft tumor formation *in vivo*. Representative xenograft tumor images **(A)**, tumor masses **(B)** and tumor volumes **(C)**. **(D,E)** Representative IHC staining of Ki67 for indicated xenograft tumors. **(F)** Representative mice images after DMBA/TPA treatment. WT = wild type (RETSATQ/Q). MUT = mutant (RETSATR/R). **(G)** The overall survival (OS) analysis for RETSAT mutation and RETSAT WT mice after DMBA/TPA induced cutaneous keratinocyte carcinoma. *p < 0.05, **p < 0.01, ***p < 0.001.

To verify the *in vivo* functional role of RETSAT during tumorigenesis, we performed both the xenograft tumor formation and DMBA/TPA induced cutaneous keratinocyte carcinoma formation assays. Five-weeks old male mice of RETSAT^R/R^ mutant and wild-type littermates were randomly divided into indicated groups, and B16 cells were injected subcutaneously (2 × 10^5^ cells/point). As expected, the subcutaneous tumors in the wild-type littermates (RETSAT^Q/Q^) were detected quickly, whereas the xenograft tumors were markedly retarded in RETSAT^R/R^ mice, which was visualized by the reduced tumor mass and volume compared to the control group (Figures 5A–C). Consistently, significant lower proliferation as measured by Ki67 immunohistochemistry (IHC) staining in the xenograft tumor sections from RETSAT^R/R^ group was detected, compared to control group (Figures 5D,E). In addition, by using the DMBA/TPA induced cutaneous keratinocyte carcinoma formation assay, better overall survival rate was observed in RETSAT^R/R^ mice compared to the littermate wild-type RETSAT^Q/Q^ mice (Figures 5F,G).

### RETSAT Q247R Mutation Inhibits Pin1 Related Signaling Pathway

**FIGURE 6:**
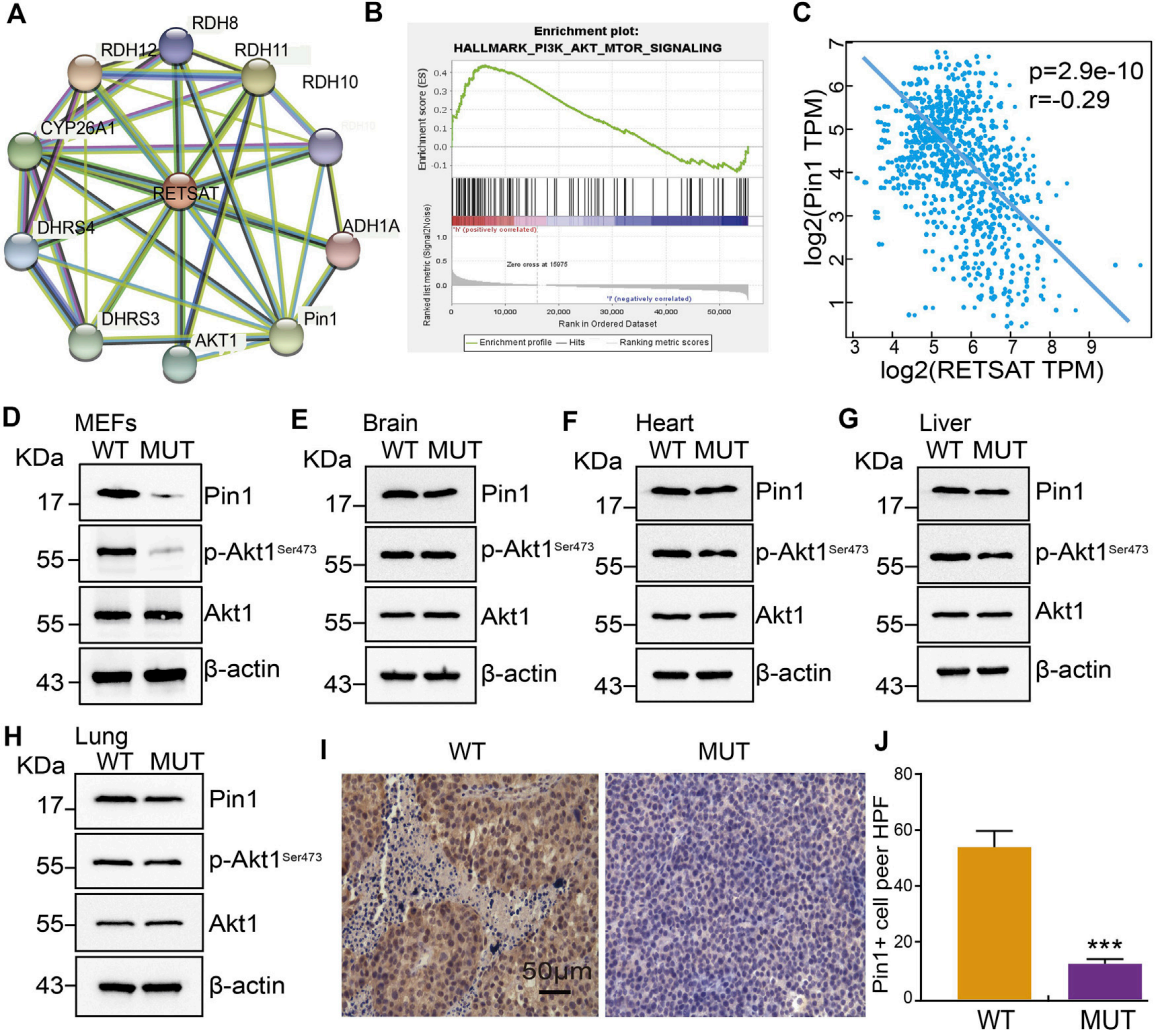
RETSAT (Q247R) mutation represses Pin1 related signaling pathway. **(A)** The protein interaction network of RETSAT was generated using STRING database. **(B)** Enrichment plots from GSEA showing the PI3K/Akt signaling pathway associated with RETSAT expression in SKCM. **(C)** The spearman correlation analysis revealed a correlation between RETSAT and Pin1 expression in TCGA-SKCM database. **(D–H)** Examining the relative protein expressions by immunoblot in MEFs **(D)**, Brain **(E)**, Heart **(F)**, Liver **(G)** and Lung **(H)** isolated from of wild-type and mutant mice, respectively. **(I–J)** Representative IHC staining of PIN1 for indicated xenograft tumors after RETSAT mutation. *p < 0.05, **p < 0.01, ***p < 0.001.

To further explore the molecular events affected by RETSAT^R/R^ mutation in SKCM, a protein-protein interaction (PPI) network for RETSAT was created using the STRING database (Szklarczyk et al., 2017) (Figure 6A). Among the interacting proteins, Pin1 and Akt1 caught our attention. The oncogenic factor PIN1 has been well demonstrated to promote the occurrence and development of various cancers (Min et al., 2016; Rustighi et al., 2017; Chen et al., 2018; Nakatsu et al., 2020; Yu et al., 2020). Previous study also documented that Pin1 inhibition using small molecule inhibitor such as ATRA or short hairpin RNA, reduces tumor growth via inhibiting PI3K/AKT signaling pathways (Sun et al., 2019). In addition, we revealed that RETSAT is involved in regulating PI3K/AKT signaling pathway by GSEA dataset analysis (Subramanian et al., 2005), suggesting that RETSAT could inhibit the oncogenic effect mediated by Pin1 in SKCM (Figure 6B).

Therefore, we decided to validate the correlation between RETSAT expression and Pin1 related signaling (Sun et al., 2019). We showed that RETSAT expression was significantly negatively correlated with Pin1 in SKCM (*r* = −0.29, *p* = 2.9e-10) (Figure 6C). To validate the signaling axis, we used various adult mouse tissues, including brain, heart, liver and lung, and uncovered that the markedly reduced Pin1 and phosphorylated Akt1 proteins in RETSAT^R/R^ background could only be detected in MEFs, liver and lung, but not in brain and heart, suggesting that the inhibitory effect could be cellular context or developmental stage dependent (Figures 6D–H). Furthermore, we revealed that RETSAT^R/R^ mutation inhibits Pin1 expressions in B16 xenograft tumors, compared to the RETSAT^Q/Q^ wild-type control groups, detected by immunohistochemistry (IHC) staining (Figures 6I,J).

## Discussion

Melanoma incidence rates have sky rocketed in the past decades, and there are recurrent somatic mutations that appear frequently in most types of melanoma (Scolyer et al., 2011; Read et al., 2016). Driving mutations have been linked to signaling pathways that regulate proliferation (BRAF, NF1 and PTEN), cellular apoptosis (TP53), and cell cycle control (CDKN2A) (Scolyer et al., 2011; Read et al., 2016). Melanoma cells quickly adapt to the immune response due to the highly mutagenic nature (Lee et al., 2016). Recent studies have demonstrated that melanoma can be efficiently overcome by treating with antibodies against PD1, PD-L1/2 and CTLA-4 (Koller et al., 2016).

Numerous studies focusing on RETSAT have demonstrated its critical role for liver metabolism (Schupp et al., 2009; Heidenreich et al., 2017). RETSAT depletion has been shown to reduce the activity of carbohydrate response element binding protein (ChREBP), a cellular hexose-phosphate sensor and inducer of lipogenesis (Heidenreich et al., 2017). Ectopic expression of RETSAT with an intact, but not a mutated, FAD/NAD dinucleotide-binding motif, increased endogenous PPARgamma transcriptional activity and promoted adipogenesis (Schupp et al., 2009). Previous results showed that all-trans retinoic acid (ATRA), one of the active metabolites of vitamin A, could successfully treat patients with acute promyelocytic leukemia (APL) (Gottardi et al., 2020). Another study uncovered that ATRA prevented the relapse of breast cancer via promoting the differentiation of cancer stem cells (Li et al., 2011). In addition, ATRA has been applied to inhibit tumor cell growth in glioblastoma (Mirani et al., 2019). 9-cis RA (9-cis-13,14-dihydroretinic acid) is a potential activator of RARs and RXRs, which has been uncovered to inhibit tumor progression (Gottardis et al., 1996; Christov et al., 2002; Karsy et al., 2010).

Peptidyl-prolyl *cis*-trans isomerase NIMA-interacting 1 (Pin1) was originally identified in 1996 (Ping Lu et al., 1996), which functions as an enzyme specifically catalyzing the isomerization of phosphorylated serine-proline or phosphorylated threonine-proline (pSer/Thr-Pro) motifs (Zhou and Lu, 2016). Emerging evidence has demonstrated that Pin1-mediated prolyl isomerization plays pivotal roles under both physiological and pathological conditions, including in human cancers (Sacktor, 2010; Tun-Kyi et al., 2011). Pin1 is aberrantly increased or constitutively activated in multiple tumors (Chen et al., 2018), and high expression of Pin1 is closely correlated to poor clinical prognosis (Zhou and Lu, 2016). Pin1 regulates the self-renewal of Cancer Stem Cells (CSCs) by maintenance the stability of Nanog, octamer-binding protein 4 (OCT4), and MYC (Nishi et al., 2011; Farrell et al., 2013). Importantly, recent study also showed that Pin1 inhibition by ATRA or short hairpin RNA, reduces cancer development by inhibiting Wnt/β-catenin and PI3K/AKT signaling pathways in gastric cancer (Zhang et al., 2019).

In this study, we showed that hypoxia adaptation selection mutant form of RETSAT^R/R^ inhibits xenograft tumor cell growth (Xu et al., 2021), and DMBA/TPA induced cutaneous keratinocyte carcinoma formation *in vivo*. However, higher expression of RETSAT was observed in gliomas (Figure 1A), suggesting that RETSAT may have differential role in the central nervous system. Multiple mutations on *RETSAT* were also identified in SKCM (Table 1), but none of them is Q247R mutation, which is more likely a gain-of-function mutation based on the *in vitro* enzymatic assay (Xu et al., 2021). Previous evidence has shown that RETSAT saturates the 13-14 double bond of all-trans-retinol to produce all-trans-13-14-dihydroretinol, a product important for vitamin A metabolism. While, all-trans retinoic acid (ATRA), the active metabolite of vitamin A and the downstream bio-product of RETSAT, has recently been used to inhibit and degrade Pin1, leading to reduced APL and triple negative breast cancer growth (Moise et al., 2004; Wei et al., 2015). Therefore, the extrinsic inhibitory function of RETSAT^R/R^ mutant might result from the increased generation of its downstream bio-products (Xu et al., 2021) or/and increased immune infiltration level (Figures 3A,B). In addition, we showed that RETSAT is closely associated with Pin1 and Akt1 by PPI network analysis, and also involved in PI3K/Akt signaling pathway by GSEA analysis (Figures 6A,B). Together with the findings that RETSAT knockdown promotes, while RETSAT overexpression inhibits, mouse B16 and fibroblast cell proliferation (Figures 4A–M), and RETSAT^R/R^ mutation reduces Pin1 and phosphorylated Akt1 protein expressions (Figures 6D–H), we hypothesized that the intrinsic inhibitory role of RETSAT could be mediated by protein-protein interaction. Therefore, to further explore the extrinsic and intrinsic mechanisms of RETSAT repressing tumor progression, the gene/protein interacting network as well as the expression profiles of the downstream bio-products, should be thoroughly characterized in the future.

Regard to our findings that RETSAT might play as a tumor suppressor in most types human cancers, we hypothesized that the somatic mutations on RETSAT in SKCM tissues mostly cause loss-of-function. Retinol has six biologically active isoforms, including all-trans, 11-cis, 13-cis, 9,13-di-cis, 9-cis, and 11,13-di-cis, with all-trans being the predominant form (Rhee and Plutzky, 2012). Exploring the potential anti-cancer roles of the precursors of vitamin A metabolism will pave a new way treating SKCM patients, especially those with advanced stages. Therefore, to improve the clinical outcome for SKCM patients, it will be critical to identify new drugs or strategy to stabilize and activate RETSAT in the future.

## Materials and Methods

### Immune Infiltration Analysis

We employed the TIMER to analyze the correlation between RETSAT expression and immune infiltration (Li et al., 2017). The TISIDB database was employed analyzing the association between RETSAT and immune regulators (immunostimulators or immunoinhibitors) (Ru et al., 2019). GEPIA and UALcan databases were employed to analyze the expression and prognosis of RETSAT in TCGA SKCM (Chandrashekar et al., 2017; Tang et al., 2017).

### Cell Culture and Reagents

Mouse embryonic fibroblasts (MEFs) were generated from RETSAT^R/R^ (mutant) or RETSAT^Q/Q^ (wild-type) mice, and B16 cells were cultured in DMEM medium (Corning) supplemented with 10% fetal bovine serum (FBS) and 1% penicillin/streptomycin. Cells were all incubated in a humidified atmosphere with 5% CO_2_ at 37°C.

### Constructs, Transfection and Lenti-Viral Infection

As described before (Xu et al., 2021), RETSAT CDS (or mutant CDS DNA fragment) was sub-cloned into pCDH-MSCV-E2F-eGFP lenti-viral vector with a 3×Flag tag at the C-terminus. Independent shRNAs targeting RETSAT mRNA were synthesized and sub-cloned into the lenti-viral vector pLKO.1 (Addgene, Cambridge, United States). Cells were transfected with indicated shRNAs or control scramble shRNA using Lipofectamine 2000 (Invitrogen), and then collected for various experiments, mouse mRETSAT-shRNA#1: GTG​GTG​TCC​TCC​CTC​CTA​CAG, mRETSAT-shRNA#2: AGC​AAT​TCC​TTG​CAC​ATA​TAA.

### Real-Time RT-PCR Assay

For Real-time RT-PCR assay, indicated cells were lysed by RNAiso Plus (Takara Bio, Beijing, China, Cat. 108-95-2). Total RNAs were extracted according to the manufacturer’s protocol, and then reverse transcribed using RT reagent Kit (Takara Bio, Beijing, China, Cat. RR047A; TIANGEN Biotech, Beijing, China, Cat. KR211-02). Real-time PCR was performed by FastStart Universal SYBR Green Master Mix (Roche, Cat. 04194194001; TIANGEN Biotech, Beijing, China, Cat. FP411-02) using an Applied Biosystems 7500 machine. The primers used in this study are: mRETSAT-qPCR-F: CCC​ATC​AAG​CAA​GGA​TCC​AA, mRETSAT-qPCR-R: ATG​GGT​ACC​AGC​GCA​GTC​A.

### Cell Proliferation

The cell proliferation assay was performed as previously described (Xu et al., 2020). For cell growth assay, indicated cells were plated into 12-well plates and the cell numbers were subsequently counted each day. For BrdU incorporation assay, indicated cells were cultured in 8-well plates for 24 h, pulsed with 10 μM BrdU (Abcam, Cat# ab142567) for 20 min, and fixed with 4% PFA (paraformaldehyde). Cells were then incubated with BrdU (Cell Signaling Technology, Cat# 5292s, dilution 1:1,000) primary antibody followed by secondary antibody detection (Abclonal, Cat# 61303, dilution 1:500). Cell nuclei were stained with DAPI (4′, 6-diamidino-2-phenylindole).

### Xenograft Tumor Formation Assay

The transgenic mice (RETSAT^R/R^) and their wild-type littermates (RETSAT^Q/Q^) were derived from C57BL/6 mouse. About 4–6 weeks old age mice were subcutaneously injected with B16 cell lines (2 × 105 cells), 3 weeks later, all mice were sacrificed. The xenograft tumors from indicated groups were harvested and weighted. The mice were monitored every other day, xenograft tumor weights and volumes were measured with a sliding caliper, and tumor volumes were calculated using the formula (L×W2)/2. All animals were kept in a SPF environment and the protocols were pre-approved and conducted under the policy of Animal care and Use Committee at the Kunming Institute of Zoology, CAS.

### DMBA/TPA Induced Cutaneous Keratinocyte Carcinoma

The assay was performed as previously described (Darido et al., 2011; Jiang et al., 2017). Briefly, 25 μg DMBA (Sigma Aldrich) in 200 µl acetone were applied to the dorsal skin after shaving. After 2 weeks, TPA (10 nmol) in 200 μl was applied to the same area twice weekly for up to 30 weeks. Skin specimens were collected 5 and 8 weeks after DMBA treatment, and when papilloma and SCC formed. The number of tumors per mouse was counted each week as palpable mass >1 mm in size.

### Western Blot

To detect the protein expressions of Pin1, Akt1 and P-Akt1, cells were lysed in IP lysis buffer, supplemented with complete protease inhibitor cocktail (Complete Mini, Roche). Indicated proteins were detected with indicated antibodies by western blot. Proteins were resolved on SDS polyacrylamide gels, and then transferred to a polyvinylidene difluoride membrane. After blocking with 5% (w/v) milk, the membrane was stained with indicated primary antibodies as follows: Pin1(Catalog number, R25374, Dilution, 1:1,000, Supplier, ZENBIO), (Akt, Catalog number, 9272, Dilution, 1:1,000, Supplier, Cell Signaling Technology), (p-S473-Akt, Catalog number, 9271, Dilution, 1:1,000, Supplier, Cell Signaling Technology), β-actin (Catalog number, 60008-1-1g, Dilution, 1:20,000, Supplier Proteintech).

### Immunohistochemistry Assay

For immunohistochemical staining, the sections were deparaffinized in xylene and rehydrated through graded ethanol. Antigen retrieval was performed for 20 min at 95°C with sodium citrate buffer (pH 6.0). After quenching endogenous peroxidase activity with 3% H_2_O_2_ and blocking non-specific binding with 1% bovine serum albumin buffer, sections were incubated overnight at 4°C with indicated primary antibodies: Pin1(Catalog number, R25374, Dilution, 1:1,000, Supplier, ZENBIO), Ki67 (Catalog number, 170, Dilution, 1:400, Supplier, NOVUS). Following several washes, the sections were treated with HRP conjugated secondary antibody for 40 min at room temperature, and stained with 3, 3-diaminobenzidine tetrahydrochloride (DAB). Slides were photographed with microscope (Olympus BX43F, Japan). The photographs were analyzed with the Image-Pro Plus 7.0 software (Media Cybernetics, Inc., Silver Spring, MD, United States).

### Statistics

All data are presented as the mean ± SEM. All experiments were performed at least three times. All analyses were performed using GraphPad Prism 7 (GraphPad Software). Two-tailed Student’s *t*-test was used for statistical analysis for experiments with two comparisons. *p*-values less than 0.05 were considered statistically significant. For all figures, *p < 0.05, **p < 0.01, ***p < 0.001.

## Data Availability

The original contributions presented in the study are included in the article/Supplementary Material, further inquiries can be directed to the corresponding authors.
