# Supplementary figures and images for: RETSAT Mutation Selected for Hypoxia Adaptation Inhibits Tumor Growth
Source: Front Cell Dev Biol. 2021 Nov 4;9:744992. doi: 10.3389/fcell.2021.744992 (PMC8601408; doi:10.3389/fcell.2021.744992)

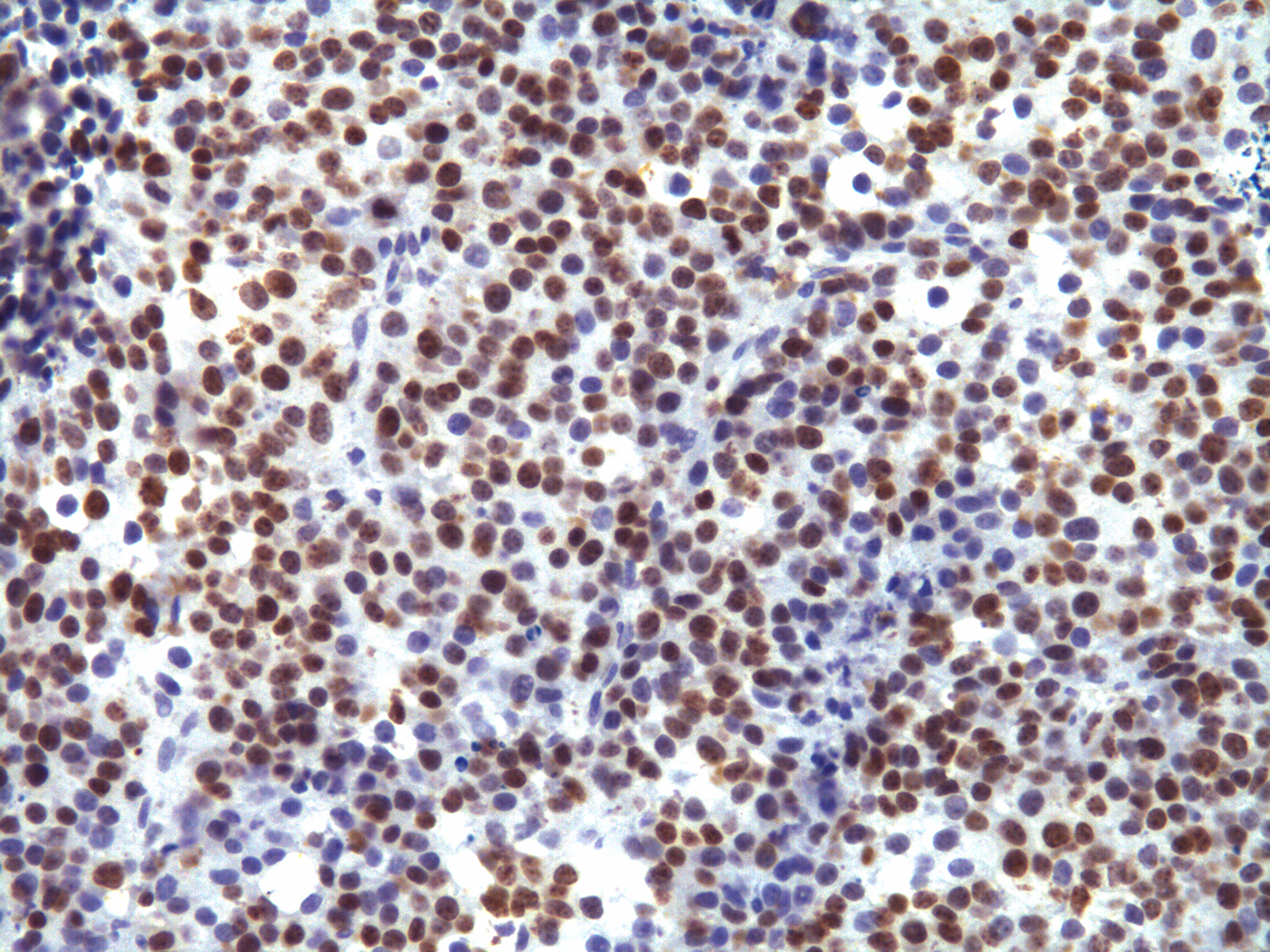

Supplement: Supplementary file 4 [file Image2.TIF]

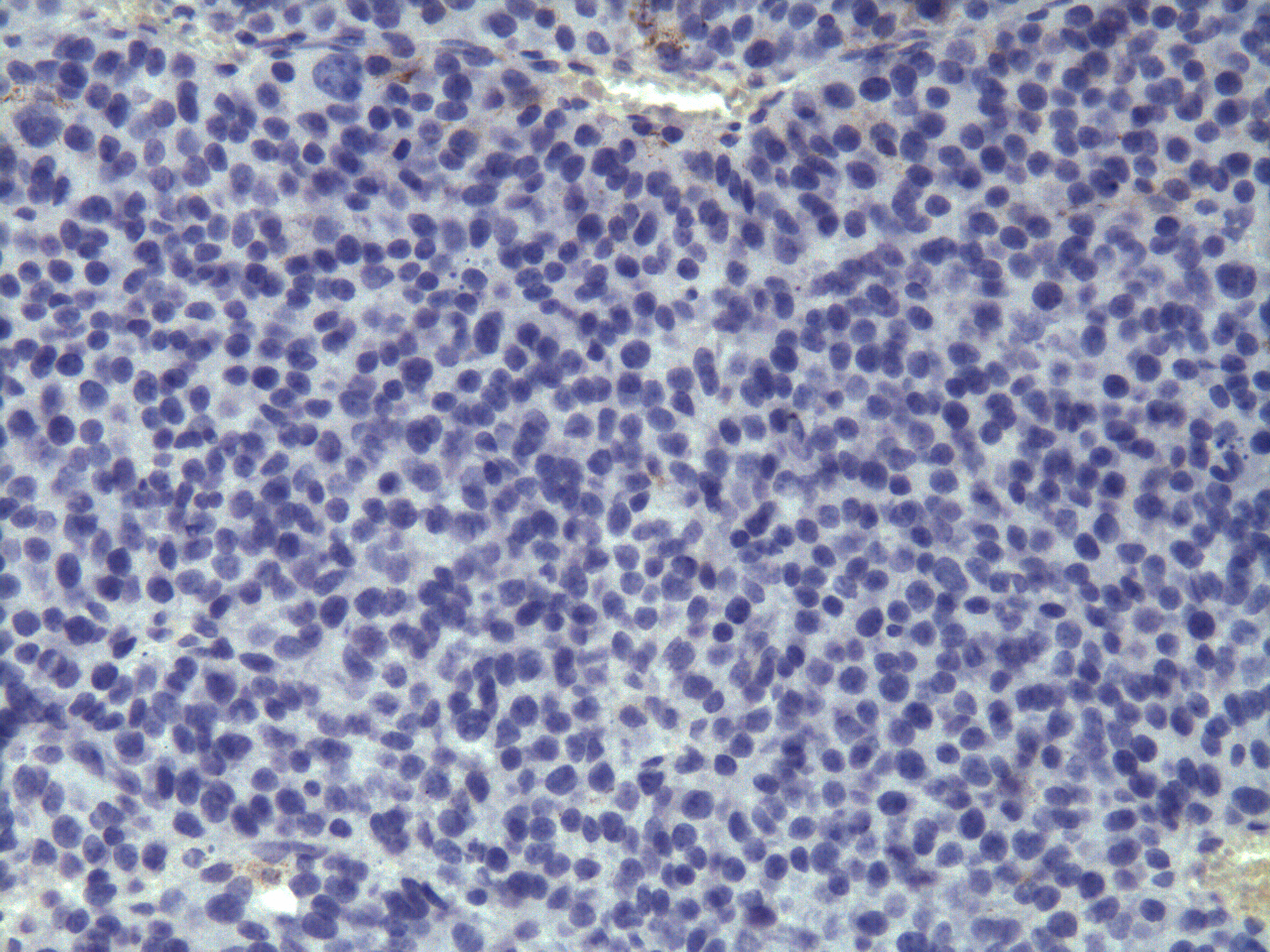

Supplement: Supplementary file 5 [file Image1.TIF]
